# A core outcome set development for a French national prospective study about the effect of mediolateral episiotomy on obstetric anal sphincter injury during operative vaginal delivery (INSTRUMODA)

**DOI:** 10.1186/s12884-021-03603-0

**Published:** 2021-03-25

**Authors:** Bertrand Gachon, Thomas Schmitz, France Artzner, Olivier Parant, Renaud De Tayrac, Guillaume Ducarme, Camille Le Ray, Anne Cécile Pizzoferrato, Charles Garabedian, Didier Riethmuller, Fabrice Pierre, Stephanie Ragot, Xavier Fritel, the GROG (Groupe de Recherche en Gynécologie Obstétrique)

**Affiliations:** 1 Poitiers University Hospital, Department of Obstetrics and Gynecology, Poitiers, France; 2 INSERM CIC-P 1402, Poitiers University Hospital, Poitiers, France; 3 Nantes University, Movement – Interactions – Performance, MIP, EA4334, Nantes, France; 4 Robert Debre Hospital, Department of Obstetrics and Gynecology, Paris, France; 5 Patients representative. CIANE, Collectif inter associatif autour de la naissance, Paris, France; 6 Paule de Viguier Maternity, Toulouse University Hospital, Toulouse, France; 7 Caremeau University Hospital, Department of Obstetrics and Gynecology, Nimes, France; 8 Centre Hospitalier Départemental de La Roche sur Yon, Department of Obstetrics and Gynecology, La Roche sur Yon, France; 9 Port Royal maternity unit, Cochin Hospital, APHP, Paris, France; 10 Caen University Hospital, Department of Obstetrics and Gynecology, Caen, France; 11 Jeanne de Flandres maternity unit, Lille University Hospital, Lille, France; 12 Grenoble University Hospital, Department of Obstetrics and Gynecology, Grenoble, France

**Keywords:** Obstetric anal sphincter injury, Operative delivery, Episiotomy, Core outcome set

## Abstract

**Background:**

We aimed at developing a core outcome and variables of interest set to investigate the effects of mediolateral episiotomy on Obstetric Anal Sphincter Injury (OASI) during and after operative delivery in nulliparous women in a large-scale one-year observational French study including 15,000 women (INSTRUMODA).

**Methods:**

A list of outcomes and variables of interest was suggested to obstetricians participating in the INSTRUMODA study using online questionnaires divided into 7 categories: the woman’s history and course of pregnancy, course of labor, modalities of operative delivery, episiotomy characteristics, immediate maternal morbidity, one-year maternal morbidity, immediate neonatal morbidity. We used a three-round DELPHI method to reach a consensus. In the first round, outcomes and variables considered as essential by 70% or more of obstetricians were included in the corpus whereas they were excluded when 70% rated them as “not important”. In the second round, non-consensual outcomes and variables were reassessed and excluded or definitively included if considered as “not important” or essential by 50% or more of the obstetricians. During the first round, obstetricians were invited to suggest new outcomes and/or variables that were then assessed in the second and third round. We used the same method to develop a core outcome and variables of interest set in a population of women in the community recruited via an association of patients. At the end of the procedure the core outcome and variables of interest sets were merged to provide the final core outcome set for the INSTRUMODA study.

**Results:**

Fifty-three obstetricians and 16 women filled out questionnaires. After the 3 rounds of Delphi procedure in each population, 74 outcomes and variables were consensually reported by obstetricians and 92 by women in the community. By mixing these two consensual corpora we reported a final consensual list of 114 variables of interest and outcomes for both obstetricians and women.

**Conclusion:**

We established a core outcome and variables of interest set among obstetricians and women in the community to investigate the association between mediolateral episiotomy and OASI during operative delivery.

**Trial registration:**

The INSTRUMODA study was registered on https://clinicaltrials.gov on June 25, 2020 (NCT04446780).

**Supplementary Information:**

The online version contains supplementary material available at 10.1186/s12884-021-03603-0.

## Background

Obstetric anal sphincter injury (OASI) is a maternal delivery complication defined as a perineal tear involving at least a superficial tear of the external anal sphincter and, at worst, an opening of the rectal mucosae [1, 2]. Such a perineal trauma is associated with significant alteration in women’s health: anal incontinence, perineal pain, dyspareunia, and postnatal depression [3]. There have been several risk factors reported for OASI occurrence, among which the most important are nulliparity and operative vaginal delivery, especially in case of forceps delivery [1–4]. When these two risk factors are associated, OASI prevalence ranging from 2 to 20% has been reported [5–8]. Even when such a high-risk situation is clearly identified, preventive strategies to avoid OASI occurrence remains disappointing.

As regards spontaneous vaginal delivery, an abundant literature reports that routine mediolateral episiotomy does not protect against OASI [9]. As regards its interest in operative vaginal delivery, the existing data are subject to considerable debate. While several retrospective studies have reported that mediolateral episiotomy has a protective effect against OASI, a small pilot randomized trial failed to identify a significant preventive effect [5–8, 10, 11]. This lack of definitive evidence means that the protective effect of mediolateral episiotomy in operative delivery remains hypothetical. Because OASI is a low frequency event and because episiotomy has its own morbidity (pain, dyspareunia, infection), systematic episiotomy may induce more harm than good [9]. There are consequently no clear recommendations about its use during operative vaginal delivery [2, 12, 13].

We have chosen to conduct a large-scale French observational study, for 1 year in more than 120 recruiting centers, with 15,000 expected inclusions of nulliparous women who will undergo operative vaginal delivery with or without mediolateral episiotomy (INSTRUMODA, NCT 04446780 https://clinicaltrials.gov). In cases of operative delivery, we will assess, the effect of mediolateral episiotomy (versus no) on OASI occurrence in cases of instrumental delivery, on immediate and one-year maternal morbidity, and on neonatal immediate morbidity. Propensity scores will be used to control for indication bias. The study is expected to start by the first trimester of 2021.

We have aimed at organizing a study that fits with both women and obstetrician’s expectations. Such an objective implies that initially, we interrogate women and professional on their expectations in view of consensually defining a corpus of endpoints. Indeed, an approach centered on both patient and professional consensual expectations is more and more often considered as a quality indicator for research projects and required by journals and funding organizations [14–16].

The main endpoint of this study was to develop a core outcome and variable of interest set on the endpoints to be addressed in the INSTRUMODA study.

## Methods

This Core Outcome Set procedure was led prospectively using an online questionnaire. The procedure that we detail precisely below followed the COS-STAD recommendations for core outcome set developments and the COMET guidelines [15, 16].

### Constitution of a scientific committee

We have organized a scientific committee to steer the INSTRUMODA study (see authors list). The 13 members of this committee are obstetricians with a specific interest in perineal trauma at childbirth, methodologist, or patient representatives *(Collectif inter associative autour de la naissance).* All the committee members were involved in the overall design of the INSTRUMODA study, including core outcome set processing.

### Identification of outcomes and variables of interest

Based on clinical or methodological experience, the scientific committee suggested a list of outcomes and variables of interest divided into 7 categories: woman’s characteristics and course of pregnancy, course of labor, modalities of operative delivery, modality of episiotomy, immediate maternal morbidity, immediate neonatal morbidity and one-year maternal morbidity. For each category, a list of outcomes and variables was consensually formulated by the scientific committee.

We chose to dichotomize the list of suggested outcomes and variables of interest with one corpus for the obstetricians (Additional file 1) and a second corpus for women in the community (Additional file 2). Our choice was justified by our considering that the understanding and interpretation of the suggestions might differ between the two samples.

We initially elaborated the list of outcomes and variables and processed the core outcome and variable of interest set for obstetricians and secondly, did the same for women in the community. At the end of the processing, the two corpuses of consensual outcomes and variables of interest were mixed to provide a final corpus of outcomes.

### Constitution of stakeholder groups

To constitute the obstetrician stakeholder group, we invited all the main investigators in all the planned recruiting centers of the INSTRUMODA study to participate in the Core outcome and variable of interest set processing. They were contacted by email and invited to participate in the consensus process. To constitute the stakeholder group composed of women in the community, we contacted volunteer women through a patient representative association *(Collectif interassociatif autour de la naissance).* Women were contacted by email and invited to participate in the consensus processing using online questionnaires in a same way as professionals. All these women had a personal birth experience, and they have a special interest about protecting mother’s health and autonomy during childbirth.

For both women and professionals, it was clearly indicated that we expected them to consensually define a list of outcomes and variables of interest that could be investigated in the INSTRUMODA study.

### Consensus processing

We defined a priori modalities of consensus processing that were comparable for both obstetricians and women. The process is based on a DELPHI method and with conformity with the COS-STAD recommendations for core outcome set developments and the COMET guidelines [15, 16].

In a first round of online questionnaires, participants were asked to attribute a level of importance to all the suggestions from the scientific committee, using a three-level scale: not important, important but not essential, essential. When at least 70% of respondents considered the item as essential, it was included in the consensual corpus. Conversely, when at least 70% of respondents considered the item as not important, it was excluded from the process. All the outcomes and variables for which the consensus was not reached were included in a second round of online questionnaires. During the first-round volunteers were asked to suggest additional outcomes and/or variables of interest for a consensus processing assessment during the second round.

In the second round, volunteers were requested to definitively attribute a level of importance to non-consensual outcomes and variables from the 1st round: important versus not important. It was clearly indicated that “important” meant that the item would be assessed in the INSTRUMODA study and that “not important” meant that it would not be assessed. When at least 50% of respondents considered the item as important, it was included in the consensual corpus; if there were less than 50%, it was excluded. During the second round respondents were also asked to attribute a level of importance to a list of new outcomes and variables of interest suggested during the first round in the same way as previously reported: not important, important but not essential, essential. These new outcomes and variables were then either included in the consensual corpus, excluded from the corpus or included in a third round of questionnaires with exactly the same methods as those applied in the 1st round.

Last, in the third round of questionnaires, respondents were asked to definitively attribute a level of importance to outcomes and variables added in the first round and not consensual in the second round. We used exactly the same methods as those applied in reported for the second round, and outcomes and variables considered by 50% or more of respondents as important were included in the consensual corpus.

At the end of the process, having obtained a core outcome and variables of interest set from both obstetricians and women in the community, we mixed these 2 corpuses to provide a final core outcome set.

### Ethical and regulatory considerations

This study involves only volunteers for anonymous online questionnaire answering, without any identifying information collection and with a total independence from any medical care. This considered, regarding the French legislation about medical research involving human person (Loi Jardé), an ethical committee approbation is not required (https://www.legifrance.gouv.fr/codes/id/LEGISCTA000032722874/2017-06-14/).

The INSTRUMODA study was registered on https://clinicaltrials.gov on June 25, 2020 (NCT 04446780). The INSTRUMODA project have been approved by an ethical committee: *Comité de Protection des Personnes Nord Ouest IV* (ID RCB: 2020-A01974-35).

## Results

### Development of a core outcome and variable of interest set in the population of obstetricians

At the time of organization of the core outcome and variables of interest set process, we planned to recruit 109 centers and an invitation to participate was sent to the main investigator of each center. There was one male midwife working in a public hospital, and 108 obstetricians. Among the 108 obstetricians 64 were males (59.3%) and 44 females (40.7%), they worked in public hospital for 92.6% (100 obstetricians) and 8 worked in a private maternity (7.4%). Each was the local volunteer to coordinate the INSTRUMODA study in its maternity and can be the head of the department but without any obligation.

#### First round

**Fig. 1:**
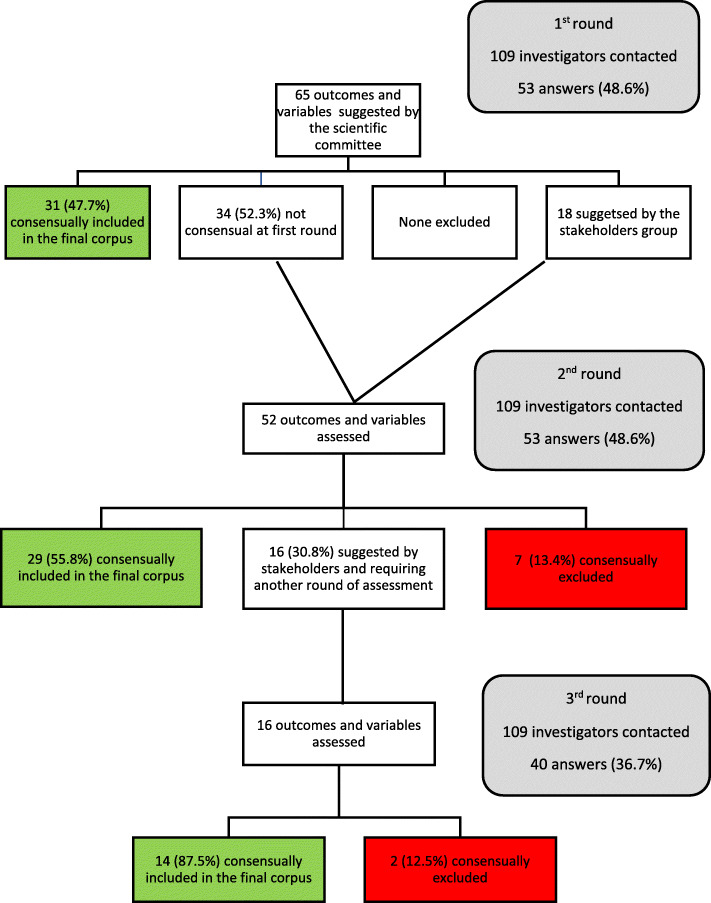
Flow Chart for the consensus process in the group of professionals

Sixty-five outcomes and variables of interest were suggested by the scientific committee and assessed by the stakeholder group. Fifty-three volunteers filled out the online questionnaire (48.6%). Thirty-one outcomes or variables were included in the final consensual corpus and none were excluded (Fig. 1). The stakeholder group suggested 18 new outcomes and variables: woman’s geographic origin, vaginismus before delivery, anal incontinence during pregnancy, maternal fever during labor, oxytocin use, number of vaginal examinations, fetal heart analysis during expulsive phase, maneuver of active delivery of the anterior arm (Couder maneuver), instrument justification, antenatal discussion about episiotomy in a birth project, experience of the practitioner performing episiotomy, analgesia for episiotomy incision, postpartum urinary retention, neonatal analgesic consumption, bone fracture, Cephalhematoma and/or subgaleal hemorrhage, wish for another pregnancy, in case of another pregnancy wish for vaginal delivery.

#### Second round

The same 109 investigators were contacted and 53 filled out the online questionnaire. Fifty outcomes and variables were assessed: 34 suggested by the scientific committee and not consensual during t the first round, 18 suggested by the stakeholder group. Twenty-nine outcomes and variables were definitively included in the corpus (Fig. 1). Seven were excluded: need for medical assistance for procreation, methods for labor induction, ultrasound assessment of fetal head station, antibioprophylaxis for operative delivery, angle of episiotomy suture from the midline, self-declared episiotomy length, length of episiotomy suture.

#### Third round

Forty of the 109 contacted investigators filled out the online questionnaire (36.7%). Sixteen outcomes and variables suggested by the stakeholder group required a final assessment. Fourteen were definitively included and 2 were excluded (number of vaginal examinations, antenatal discussion about episiotomy within a birth project) (Fig. 1).

### Development of a core outcome and variable of interest set in the population of women in the community

Twenty-four women were contacted and requested to fill out the 1st round of online questionnaire.

#### First round

**Fig. 2:**
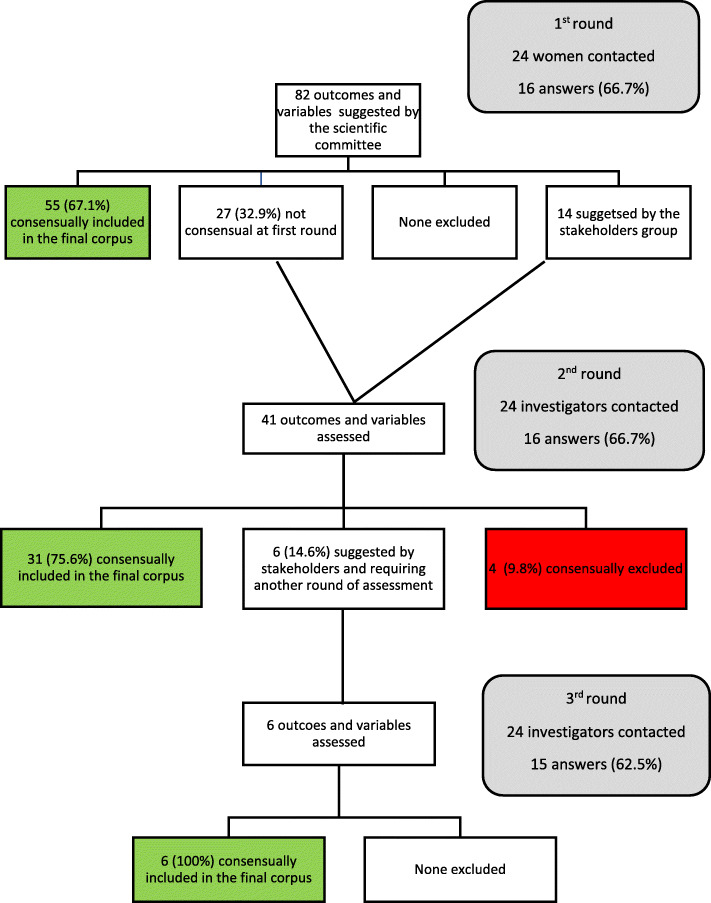
Flow Chart for the consensus process in the group of women

Sixteen of the 24 women contacted filled out the questionnaire (66.7%). Out of the 82 outcomes and variables suggested by the scientific committee, 55 were consensually included in the final corpus and none were excluded (Fig. 2). The stakeholder group suggested 14 new outcomes and variables: personal history of depression, self-rated anxiety before delivery, self-rated physical fatigue before operative delivery, self-rated psychological fatigue before operative delivery, self-rated concern immediately before operative delivery, attempt at manual fetal rotation, change of maternal position during the expulsive phase before deciding on an operative delivery, analgesia for episiotomy incision, analgesia for episiotomy reparation, difficulty of movement after delivery, self-rated level of understanding about interventions for the delivery, postnatal vaginismus, wish to prepare a birth project in case of another pregnancy, neonate hospitalization in a unit different from that the mother.

#### Second round

The same 24 women were contacted and 16 filled out the online questionnaire (66.7%). Forty-one outcomes and variables of interest were assessed: 27 for a second assessment and the 14 suggested by the stakeholder group. Thirty-one outcomes were consensually included in the final corpus and 4 were excluded: women’s geographic origin, maternal smoking, medical assistance for procreation, hour of birth (Fig. 2).

#### Third round

The same 24 women were contacted and 15 filled out the online questionnaire (62.5%). Six outcomes and variables suggested by the stakeholder group that were not consensual at the previous round were assessed and all were definitively included in the consensual corpus (Fig. 2).

### Constitution of the final core outcome and variable of interest set

All in all, 74 outcomes were consensually considered as important by obstetricians and 92 by women in the community. After mixing them in a single corpus, there resulted a corpus of 114 consensual outcomes and variables of interest (Table 1).

**Table 1:**
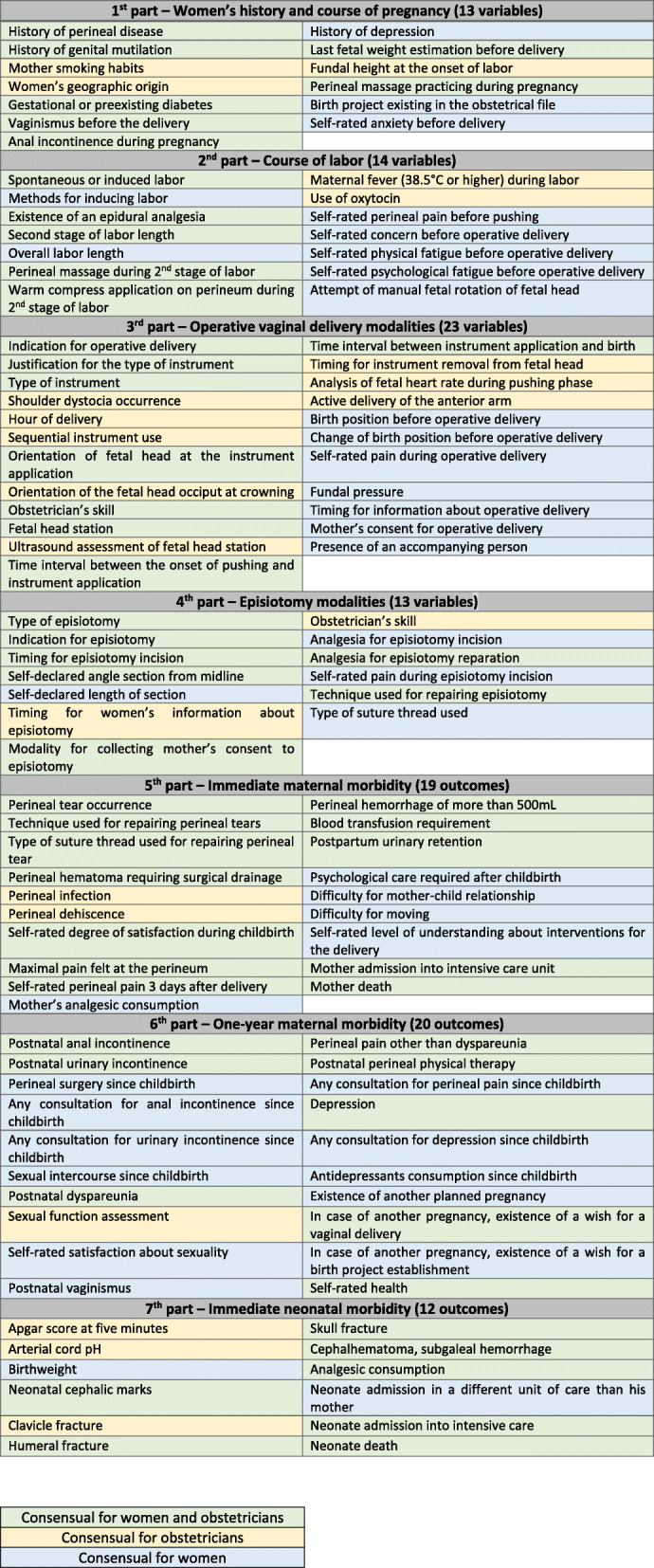
Final core outcome and variable of interest set

## Discussion

### Main findings

We developed a core outcome and variable of interest set that will be used in the INSTRUMODA study to assess the effect of mediolateral episiotomy on OASI occurrence during operative vaginal delivery in nulliparous women. A list of 114 outcomes and variables divided into 7 categories was consensually included by obstetricians and women in the community: women’s history and course of pregnancy, course of labor, modalities of operative delivery, episiotomy, immediate maternal morbidity, one-year maternal morbidity and immediate neonatal morbidity.

### Strengths and limitations

The first strength of this study is that it is the first consensual statement of outcomes and variables of interest for a research protocol investigating OASI, episiotomy and morbidity associated with operative vaginal delivery. This first report offers a possibility of providing further study protocols on this theme as close as possible to the expectations of both women and professionals.

Second, our process is in accordance with both the COS-STAD recommendations for core outcome set developments and the COMET guidelines (an indicator of reliability) [15, 16]. The most important points characterizing these guidelines are that: their scope is clearly specified, the stakeholder group included both healthcare professionals and patients, the initial list of suggested outcomes or variables of interest took both healthcare and patient views into consideration, the scoring process and consensus definition were described a priori, as were the criteria for including/dropping items [15, 16].

One limitation of our study is the fact that we developed a national core outcome and variable of interest set. This is in accordance with the aim of having it applied in a nationwide French observational study; as regards this objective, in France its interpretation should remain unrestricted. Nevertheless, extrapolation of this core outcome and variable of interest set for potential further study in another country might be limited insofar as it might not deal with medical or societal considerations specific to that country. Manual perineal protection is an excellent example considering that in French maternities all deliveries are performed with a “hands-on approach” and in French obstetrical and midwives’ schools, only the hands-on approach is learned. So much said, our core outcome and variable of interest set reports a minimal list of outcomes and variables that should be addressed. Any specific considerations due to specific practices or specific populations may easily be integrated to this list.

Another limitation is that our corpus includes an important number of outcomes and variables. When responding to the survey, the stakeholders were aware that the INSTRUMODA study will involve 15,000 women within 130 maternities and so will be one of the largest prospective cohort about operative vaginal delivery so far. It was requested to the stakeholders to specifically focus on the assessment of the hypothetic association between mediolateral episiotomy and obstetric anal sphincter injury. Nevertheless, it is likely that both women in the community and professionals have included outcomes and variables of interest that should be addressed in the study to allow future secondary analysis in this cohort (for example for other maternal morbidity associated with operative vaginal delivery). This may explain the high number of outcomes and variables of interest considered.

Finally, the response rate in the group of professionals is approximatively 50% for the first and the second round. This is usual for online surveys especially when there are several rounds of questionnaires with 40 to 70% of response rate reported in the literature. This low rate of answer might be related to the absence of obligation to answer to the consensus process to participate in the INSTRUMODA study. It is likely that if a complete fulfillment of the questionnaire had been required for an effective participation into the study, the number of answers would have been higher.

### Interpretation

Reporting both women’s and professional’s expectations appears essential to this research theme. Indeed, the question of episiotomy during childbirth is a highly debated topic of interest for both women and obstetricians. Some consider that episiotomy reduces the risk of OASI at childbirth, whereas others consider it as a form of sexual mutilation or violence against women [17]. However, that may be, women are asking for better information about this intervention and for greater involvement in the medical decisions related to their giving birth. The British Supreme Court recently held that in order to be able to make autonomous decisions about how to give birth, women have a right to information about any material risk [18]. A consensual approach to definition of the outcome of interest will improve the efficacy of studies on this theme, bringing original data providing women with the information that they are requesting, individualizing this information and thereby so improving our counseling about childbirth.

Some of the variables and outcomes from our corpus might be considered as difficult to assess or address properly, especially for some variables about operative vaginal delivery or episiotomy modalities (timing for instrument removal from the fetal head, self-declared angle section for episiotomy). We anticipated this point considering that in the INSTRUMODA a specific form about the delivery characteristic will be implemented in the recruiting maternities that will contains all the variables of the corpus (for operative vaginal delivery and episiotomy modalities) and it will be requested from the obstetrician to fulfill it immediately after the delivery. With this approach we hope to collect high quality data.

It is interesting to note that most of the suggested outcomes or variables were considered as important by both women and obstetricians, a finding suggesting that the scientific committee’s perception of these two population’s expectations was close to what they really are. The most important elements brought to light by the core outcome and variable of interest set process were women’s imperative need to improve communication and to further investigate psychological aspects: timing and modality of consent for both operative delivery and episiotomy decisions, evaluation of mental attitude and causes for concern, level of understanding etc.… This is in accordance with increasing requests for information and the above-mentioned principle of respect for individual autonomy. This reflection confirms the validity of our results, which are in congruence with the current societal debate about the autonomy of women to make decisions about childbirth.

The few points of discordance between the women’s and the obstetrician’s process were: women’s geographic origin, maternal smoking, episiotomy characteristics (length and angle), number of vaginal examinations, hour of birth. For the first two reported outcomes, this discordance reflects the debate that exists in the literature as to whether these factors should be considered as risk factors of OASI [2–4]. As regards the discordance concerning the collection of episiotomy characteristics, we can hypothesize that these items were more frequently considered by obstetricians because they might be viewed as an evaluation of their practices (whether or not the episiotomies they performed were satisfactory); in some instances, they did not wish to investigate these outcomes. The episiotomy technique is indeed important, given that to be protective a mediolateral episiotomy should be at least at 45° from the midline after suture, which implies a section at 60° minimum [1, 2, 13]. This point is more often considered as important by women, insofar as they who probably consider that if they have to undergo an episiotomy, they prefer it to be extended as little as possible. Another hypothesis is that obstetricians may have found it difficult to collect accurate data about the characteristics of episiotomy. Indeed, angle section is self-declared, producing low fidelity information along with the self-declared length. This observation is supported by a French study reporting that a number of episiotomies are not performed as recommended by international guidelines [1, 2, 13, 19]. Both suggestions “self-declared angle section from midline” and “measured angle section from midline” were assessed through the consensus process and only “self-declared angle of episiotomy” was considered as important. It is likely that the number of vaginal examinations is more often considered by women as an important outcome because they are the ones concerned with these examinations and the associated discomfort, whereas the obstetrician’s intervention is focused on the end of the labor. The hour of birth might be a marker of organizational constraints (in some maternity units, obstetricians are not permanently present during the night) that may affect childbirth management, and this is likely to be more obvious for professionals than for women. With this core outcome and variable of interest set, we expect to provide original data through the INSTRUMODA study, which will be the largest prospective cohort of operative vaginal delivery in nulliparous women. We believe that our results might improve the effectiveness of international guidelines about OASI and operative delivery, which have been disappointing regarding preventive strategies and information to deliver to women.

## Conclusion

We have developed a core outcome and variable of interest set of 114 consensual outcomes for both professionals and women in order to investigate the effect of mediolateral episiotomy on OASI occurrence during operative vaginal delivery in nulliparous women. This core outcome and variable of interest set will be applied in the INSTRUMODA study, which is a prospective nationwide cohort on operative vaginal delivery in nulliparous, for which 15,000 inclusions are expected for 1 year.

## Supplementary Information

**Additional file 1.** Online questionnaire for the obstetricians’ stakeholder group.

**Additional file 2.** Online questionnaire for the women in the community stakeholder group.

## Data Availability

The datasets generated and analyzed during the current study are available from the corresponding author on reasonable request.

## Abbreviations

GROG: French research group in obstetrics and gynecology (*Groupe de Recherche en Obstétrique et Gynécologie)*
OASI: Obstetric Anal Sphincter Injury
