# Supplementary material for: A core outcome set development for a French national prospective study about the effect of mediolateral episiotomy on obstetric anal sphincter injury during operative vaginal delivery (INSTRUMODA)
Source: BMC Pregnancy Childbirth. 2021 Mar 25;21:251. doi: 10.1186/s12884-021-03603-0 (PMC7993449; doi:10.1186/s12884-021-03603-0)
Supplement: Supplementary file 2 — Additional file 2. Online questionnaire for the women in the community stakeholder group. [file 12884_2021_3603_MOESM2_ESM.docx]

**Additional File 2**

**INSTRUMODA – Core outcome and variable of interest set**

**Online questionnaire for the women in the community stakeholder group**

**FIRST ROUND**

**Women’s history and course of pregnancy**

Regarding the INSTRUMODA project, could you indicate the level of importance for these suggestions about women’s history and course of pregnancy?

|  | Not important | Important but not essential | Essential |
| --- | --- | --- | --- |
| History of perineal disease | O | O | O |
| Vaginismus before the delivery | O | O | O |
| Anal incontinence during pregnancy | O | O | O |
| Women’s geographic origin | O | O | O |
| History of genital mutilation | O | O | O |
| Mother smoking habits | O | O | O |
| Medically assisted pregnancy | O | O | O |
| Gestational or preexisting diabetes | O | O | O |
| Last fetal weight estimation before delivery | O | O | O |
| Perineal massage practicing during pregnancy | O | O | O |
| Birth project existing in the obstetrical file | O | O | O |

Is there any additional information in this thematic that you consider important to collect in the INSTRUMODA study?

**Course of labor**

Regarding the INSTRUMODA project, could you indicate the level of importance for these suggestions about the course of labor?

|  | Not important | Important but not essential | Essential |
| --- | --- | --- | --- |
| Spontaneous or induced labor | O | O | O |
| Methods for inducing labor | O | O | O |
| Existence of an epidural analgesia | O | O | O |
| Overall labor length | O | O | O |
| Second stage of labor length (time interval between full dilatation and birth) | O | O | O |
| Perineal massage during 2^nd^ stage of labor | O | O | O |
| Warm compress application on perineum during 2^nd^ stage of labor | O | O | O |
| Self-rated perineal pain before pushing | O | O | O |

Is there any additional information in this thematic that you consider important to collect in the INSTRUMODA study?

**Operative vaginal delivery modalities**

Regarding the INSTRUMODA project, could you indicate the level of importance for these suggestions about the course of labor?

|  | Not important | Important but not essential | Essential |
| --- | --- | --- | --- |
| Hour of delivery | O | O | O |
| Birth position before operative delivery | O | O | O |
| Indication for operative delivery | O | O | O |
| Orientation and station of the fetal head | O | O | O |
| Type of instrument | O | O | O |
| Justification for the type of instrument | O | O | O |
| Obstetrician’s skill | O | O | O |
| Time interval between the onset of pushing and instrument application | O | O | O |
| Time interval between instrument application and birth | O | O | O |
| Self-rated pain during operative delivery | O | O | O |
| Fundal pressure | O | O | O |
| Timing for information about operative delivery | O | O | O |
| Mother’s consent for operative delivery | O | O | O |
| Presence of an accompanying person | O | O | O |

Is there any additional information in this thematic that you consider important to collect in the INSTRUMODA study?

**Episiotomy modalities**

Regarding the INSTRUMODA project, could you indicate the level of importance for these suggestions about episiotomy modalities?

|  | Not important | Important but not essential | Essential |
| --- | --- | --- | --- |
| Type of episiotomy | O | O | O |
| Self-declared angle section from midline | O | O | O |
| Self-declared length of section | O | O | O |
| Indication for episiotomy | O | O | O |
| Timing for episiotomy incision | O | O | O |
| Modality for collecting mother’s consent to episiotomy | O | O | O |
| Self-rated pain during episiotomy incision | O | O | O |
| Technique used for repairing episiotomy | O | O | O |
| Type of suture thread used | O | O | O |

Is there any additional information in this thematic that you consider important to collect in the INSTRUMODA study?

**Immediate maternal morbidity**

Regarding the INSTRUMODA project, could you indicate the level of importance for these suggestions about immediate maternal morbidity?

|  | Not important | Important but not essential | Essential |
| --- | --- | --- | --- |
| Perineal tear occurrence | O | O | O |
| Technique and thread used for repairing perineal tears | O | O | O |
| Perineal hemorrhage of more than 500ml | O | O | O |
| Blood transfusion requirement | O | O | O |
| Perineal hematoma requiring surgical drainage | O | O | O |
| Perineal infection | O | O | O |
| Mother admission into intensive care unit | O | O | O |
| Maximal pain felt at the perineum | O | O | O |
| Mother’s analgesic consumption | O | O | O |
| Postpartum urinary retention | O | O | O |
| Self-rated perineal pain 3 days after delivery | O | O | O |
| Self-rated degree of satisfaction during childbirth | O | O | O |
| Psychological care required after childbirth | O | O | O |
| Difficulty for mother-child relationship | O | O | O |
| Mother death | O | O | O |
|  |  |  |  |

Is there any additional information in this thematic that you consider important to collect in the INSTRUMODA study?

**One-year maternal morbidity**

Regarding the INSTRUMODA project, could you indicate the level of importance for these suggestions about one-year maternal morbidity?

|  | Not important | Important but not essential | Essential |
| --- | --- | --- | --- |
| Postnatal anal incontinence | O | O | O |
| Postnatal urinary incontinence | O | O | O |
| Postnatal perineal physical therapy | O | O | O |
| Perineal surgery since childbirth | O | O | O |
| Any consultation for anal incontinence since childbirth | O | O | O |
| Any consultation for urinary incontinence since childbirth | O | O | O |
| Any consultation for perineal pain since childbirth | O | O | O |
| Any consultation for depression since childbirth | O | O | O |
| Antidepressants consumption since childbirth | O | O | O |
| Perineal pain other than dyspareunia | O | O | O |
| Dyspareunia | O | O | O |
| Sexual intercourse since childbirth | O | O | O |
| Self-rated satisfaction about sexuality | O | O | O |
| Self-rated health | O | O | O |
| Depression | O | O | O |
| Existence of another planned pregnancy | O | O | O |
| In case pf another pregnancy, existence of a wish for a vaginal delivery | O | O | O |

Is there any additional information in this thematic that you consider important to collect in the INSTRUMODA study?

**Immediate neonatal morbidity**

Regarding the INSTRUMODA project, could you indicate the level of importance for these suggestions about immediate neonatal morbidity?

|  | Not important | Important but not essential | Essential |
| --- | --- | --- | --- |
| Neonatal cephalic marks | O | O | O |
| Hematoma on the head (Cephalhematoma, subgaleal hemorrhage) | O | O | O |
| Analgesics consumption (neonatal pain) | O | O | O |
| Birthweight | O | O | O |
| Skull fracture | O | O | O |
| Humeral (arm) fracture | O | O | O |
| Neonate admission into intensive care | O | O | O |
| Neonate death | O | O | O |

Is there any additional information in this thematic that you consider important to collect in the INSTRUMODA study?

**SECOND ROUND**

**Women’s history and course of pregnancy**

Regarding the INSTRUMODA project, could you indicate if these variables or outcomes should be addressed in the study (Important) or should not be addressed (Not important)?

|  | Not important | Important |
| --- | --- | --- |
| History of perineal disease | O | O |
| Vaginismus before the delivery | O | O |
| Women’s geographic origin | O | O |
| Mother smoking habits | O | O |
| Medically assisted pregnancy | O | O |
| Gestational or preexisting diabetes | O | O |
| Last fetal weight estimation before delivery | O | O |
| Perineal massage practicing during pregnancy | O | O |
| Birth project existing in the obstetrical file | O | O |

Regarding the INSTRUMODA project, could you indicate the level of importance for these suggestions about women’s history and course of pregnancy?

|  | Not important | Important but not essential | Essential |
| --- | --- | --- | --- |
| History of depression | O | O | O |
| Self-rated anxiety before delivery | O | O | O |

**Course of labor**

Regarding the INSTRUMODA project, could you indicate if these variables or outcomes should be addressed in the study (Important) or should not be addressed (Not important)?

|  | Not important | Important |
| --- | --- | --- |
| Overall labor length | O | O |
| Second stage of labor length (time interval between full dilatation and birth) | O | O |
| Perineal massage during 2^nd^ stage of labor | O | O |
| Warm compress application on perineum during 2^nd^ stage of labor | O | O |
| Self-rated perineal pain before pushing | O | O |

Regarding the INSTRUMODA project, could you indicate the level of importance for these suggestions about the course of labor?

|  | Not important | Important but not essential | Essential |
| --- | --- | --- | --- |
| Self-rated physical fatigue before operative delivery | O | O | O |
| Self-rated psychological fatigue before operative delivery | O | O | O |
| Self-rated concern before operative delivery | O | O | O |
| Attempt of manual fetal rotation of fetal head | O | O | O |

**Operative vaginal delivery modalities**

Regarding the INSTRUMODA project, could you indicate if these variables or outcomes should be addressed in the study (Important) or should not be addressed (Not important)?

|  | Not important | Important |
| --- | --- | --- |
| Hour of delivery | O | O |
| Obstetrician’s skill | O | O |

Regarding the INSTRUMODA project, could you indicate the level of importance for these suggestions about the operative vaginal delivery modalities?

|  | Not important | Important but not essential | Essential |
| --- | --- | --- | --- |
| Change of birth position before operative delivery | O | O | O |

**Episiotomy modalities**

Regarding the INSTRUMODA project, could you indicate if these variables or outcomes should be addressed in the study (Important) or should not be addressed (Not important)?

|  | Not important | Important |
| --- | --- | --- |
| Self-declared angle section from midline | O | O |
| Technique used for repairing episiotomy | O | O |
| Type of suture thread used | O | O |

Regarding the INSTRUMODA project, could you indicate the level of importance for these suggestions about episiotomy modalities?

|  | Not important | Important but not essential | Essential |
| --- | --- | --- | --- |
| Analgesia for episiotomy incision | O | O | O |
| Analgesia for episiotomy reparation | O | O | O |

**Immediate maternal morbidity**

Regarding the INSTRUMODA project, could you indicate if these variables or outcomes should be addressed in the study (Important) or should not be addressed (Not important)?

|  | Not important | Important |
| --- | --- | --- |
| Technique and thread used for repairing perineal tears | O | O |

Regarding the INSTRUMODA project, could you indicate the level of importance for these suggestions about immediate maternal morbidity?

|  | Not important | Important but not essential | Essential |
| --- | --- | --- | --- |
| Difficulty for moving | O | O | O |
| Self-rated level of understanding about interventions for the delivery | O | O | O |

**One-year maternal morbidity**

Regarding the INSTRUMODA project, could you indicate if these variables or outcomes should be addressed in the study (Important) or should not be addressed (Not important)?

|  | Not important | Important |
| --- | --- | --- |
| Postnatal perineal physical therapy | O | O |
| Self-rated satisfaction about sexuality | O | O |
| Self-rated health | O | O |
| Depression | O | O |
| Existence of another planned pregnancy | O | O |

Regarding the INSTRUMODA project, could you indicate the level of importance for these suggestions about one-year maternal morbidity?

|  | Not important | Important but not essential | Essential |
| --- | --- | --- | --- |
| Postnatal vaginismus | O | O | O |
| In case of another pregnancy, existence of a wish for a birth project establishment | O | O | O |

**Immediate neonatal morbidity**

Regarding the INSTRUMODA project, could you indicate if these variables or outcomes should be addressed in the study (Important) or should not be addressed (Not important)?

|  | Not important | Important |
| --- | --- | --- |
| Hematoma on the head (Cephalhematoma, subgaleal hemorrhage) | O | O |
| Birthweight | O | O |

Regarding the INSTRUMODA project, could you indicate the level of importance for these suggestions about immediate neonatal morbidity?

|  | Not important | Important but not essential | Essential |
| --- | --- | --- | --- |
| Neonate admission in a different unit of care than his mother | O | O | O |

**THIRD ROUND**

**Women’s history and course of pregnancy**

Regarding the INSTRUMODA project, could you indicate if these variables or outcomes should be addressed in the study (Important) or should not be addressed (Not important)?

|  | Not important | Important |
| --- | --- | --- |
| History of depression | O | O |
| Self-rated anxiety before delivery | O | O |

**Course of labor**

Regarding the INSTRUMODA project, could you indicate if these variables or outcomes should be addressed in the study (Important) or should not be addressed (Not important)?

|  | Not important | Important |
| --- | --- | --- |
| Attempt of manual fetal rotation of fetal head | O | O |

**One-year maternal morbidity**

Regarding the INSTRUMODA project, could you indicate if these variables or outcomes should be addressed in the study (Important) or should not be addressed (Not important)?

|  | Not important | Important |
| --- | --- | --- |
| Postnatal vaginismus | O | O |
| In case of another pregnancy, existence of a wish for a birth project establishment | O | O |

**Immediate neonatal morbidity**

Regarding the INSTRUMODA project, could you indicate if these variables or outcomes should be addressed in the study (Important) or should not be addressed (Not important)?

|  | Not important | Important |
| --- | --- | --- |
| Neonate admission in a different unit of care than his mother | O | O |
